# Early Sowing Approach for Developing Climate Resilient Maize: Cold Stress Impact on Germination of Adapted Inbred Lines with High Nutritive Value

**DOI:** 10.3390/plants14162540

**Published:** 2025-08-15

**Authors:** Marija Kostadinović, Mirjana Milovanović, Ana Nikolić, Ksenija Marković, Jelena Vukadinović, Jelena Vančetović, Dragana Ignjatović Micić

**Affiliations:** 1 Laboratory for Molecular Genetics and Physiology, Maize Research Institute “Zemun Polje”, Slobodana Bajića 1, 11185 Belgrade, Serbia; mmilovanovic@mrizp.rs (M.M.); anikolic@mrizp.rs (A.N.); jmesarovic@mrizp.rs (J.V.); idragana@mrizp.rs (D.I.M.); 2 Seed Testing Laboratory, Maize Research Institute “Zemun Polje”, Slobodana Bajića 1, 11185 Belgrade, Serbia; kmarkovic@mrizp.rs; 3 Group for Maize Breeding, Male Sterility and Dihaploids, Maize Research Institute “Zemun Polje”, Slobodana Bajića 1, 11185 Belgrade, Serbia; vjelena@mrizp.rs

**Keywords:** cold stress, germination, protein quality, secondary metabolites

## Abstract

In temperate regions, early sowing of high nutritive genotypes could support maize production sustainability by avoiding warming-related unfavorable environment conditions during flowering. Seven standard maize (SM) lines and their nine quality protein maize (QPM) counterparts were evaluated for cold tolerance during germination. Cold stress (13°/6 °C) was applied for five days, after a 48 h imbibition period under optimal temperature (25°/22 °C). Germination, physiological parameters, and some primary and secondary metabolites in the seeds were analyzed. No significant differences (*p* > 0.05) were observed in cold tolerance between SM and QPM. Cold stress significantly reduced germination energy (SM-*p* < 0.05, QPM-*p* < 0.001) and physiological traits (*p* < 0.001), with shoot traits being most severely affected. The potentially high impact of gallic (*p* < 0.001), protocatechuic (*p* < 0.05), and *p*-coumaric (*p* < 0.001) acids on germination under stress and negative effect of lutein + zeaxhantin and β-cryptoxhantin (*p* < 0.05) on root length was revealed. Among all lines, L3QPM excelled under stress, with unchanged germination energy and the lowest fold change in vigor indices (0.35 for VI1, 0.45 for VI2). Also, β + γ-tocopherol and gallic and caffeic acids were significantly higher (*p* < 0.05) compared to its SM original. Lines L1QPM2, L3QPM, and L7QPM, combining improved nutritional quality with high cold tolerance, will be incorporated in further early sowing research and breeding programs.

## 1. Introduction

Maize is the most important global crop in terms of total production and food security. Globally, maize (dry grain) is primarily used as feed (56% of production), while approximately 20% is allocated for non-food uses, and only 13% is directly consumed as food [1,2]. Nevertheless, maize remains staple food in many countries of tropical and sub-tropical regions. While it serves as a good source of calories, diets heavily reliant on maize can lead to malnutrition due to its low content of essential nutrients. The primary nutritional limitations in maize-based diets include low levels of essential amino acids lysine and tryptophan, as well as micronutrients such as vitamin A, zinc (Zn), and iron (Fe). To address these deficiencies, efforts in bio-fortification (nutritional improvement) have led to the development and production of improved maize varieties such as Quality Protein Maize (QPM), which is enriched with lysine and tryptophan. QPM was initially developed to combat malnutrition in developing regions of sub-Saharan Africa, Latin America, and South Asia [2]. It was shown that regular consumption of QPM can reduce the symptoms of illnesses caused by tryptophan and lysine deficiencies [3,4]. Although QPM is of tropical origin and its adaptation to temperate regions is frequently hampered by the retained exotic germplasm, successful adaptation of several QPM varieties has been reported [5,6,7,8]. QPM is of great interest for broiler and other monogastric animal feeds, as lysine and tryptophan are among the major limiting amino acids for their growth and development. The inclusion of QPM can improve animal performance and positively affect carcass characteristics, and thus contribute to the increasing global demand for high-quality meat production [9].

However, maize has been recognized as the crop most vulnerable to climate change, facing threats from increased and extreme temperatures, unpredictable rainfall patterns, and elevated atmospheric CO_2_ levels. Projected net warming-related losses range from 12% (with a 4.6 °C increase in maize cropland) to as much as 30% (with a 5 °C increase) by 2069–2099 [10]. Recognizing the urgent need to mitigate these negative impacts, the Food and Agriculture Organization of the United Nations (FAO) developed the FAO Strategic Framework 2022–2031, which promotes the adoption of innovative practices solutions aimed at enhancing climate resilience, adaptation, and mitigation within sustainable agrifood systems [11]. Key priorities of the framework include improving production efficiency and nutritional outcomes. Also, it is considered that the climate change impacts on crop production can be substantially reduced through adaptation of cropping systems [12] and the introduction of better-adapted crop varieties [13]. In this context, one promising adaptation strategy for maize in temperate regions involves shifting the sowing date forward—sowing early maturity cold-tolerant genotypes at sub-optimal temperatures below 15 °C. Adjusting sowing date holds significant potential to increase crop productivity by aligning the crop growing period with favorable environmental conditions such as precipitation patterns, frost and drought risk, and pests or diseases incidence [14,15,16]. Potential benefits of early sowing strategy include avoidance of heat peak stress during flowering and grain filling, improved water use efficiency through utilization of early season moisture and optimized solar radiation capture [17]. The effectiveness of this approach is often enhanced when combined with other practices, such as choosing appropriate stress-tolerant cultivars and cropping management practices. However, the impact of sowing date adjustments on crop production is strongly dependent on cultivar selection and local environmental conditions [17,18,19].

Developing climate-resilient, bio-fortified maize varieties can simultaneously enhance production sustainability and strengthen food security. At the Maize Research Institute Zemun Polje (MRIZP), a program to develop QPM counterparts of commercial hybrids resulted in six adapted hybrids with improved protein quality (designated as ZPQPM). Although early sowing of these hybrids could contribute to food security through providing high protein quality maize with sustainable yield under climate change, shifting sowing date for ten days to two weeks forward increases the risk of seedlings being exposed to several days of cold spells. As a crop of tropical and sub-tropical origin, maize is highly susceptible to cold stress throughout its life cycle, in both vegetative and reproductive growth stages. However, early vegetative stages VE to V3–V4 (germination and seedling emergence) are the most susceptible stages. Selection of cold-tolerant genotypes is therefore essential for successful early sowing adaptation, because negative effects of low temperatures include low germination and emergence rate and impaired cell survival and division, photosynthesis, and water transport—finally ending in reduced plant growth and lower productivity [20]. Overall plant development and yield sustainability strongly depends on maintenance of seed and germination-related traits (such as seed vigor, seedling emergence rate and uniformity, early root development, and architecture) under the cold stress. Hence, the main objective of the presented study was to assess the potential of parental lines of ZPQPM hybrids for early sowing by evaluating their cold tolerance during germination, as well as to determine any difference in the level of cold tolerance between ZPQPM and their original (SM—standard maize) lines. Germination parameters, seedling root and shoot morphological and physiological parameters (length, fresh and dry weight), and vigor indices VI1 and VI2 of both ZPQPM and their original SM lines were determined under optimal temperature and cold stress conditions. Additionally, biochemical analyses of primary and secondary metabolites of the seeds were performed to confirm their nutritional value and to assess their potential impact on germination and seedling growth and development. Based on the statistical analysis of the results, ZPQPM genotypes for further studies on cold tolerance under controlled and field conditions were identified.

## 2. Results

### 2.1. Seed Biochemical Analyses

#### 2.1.1. Protein and Tryptophan Content

**Figure 1:**
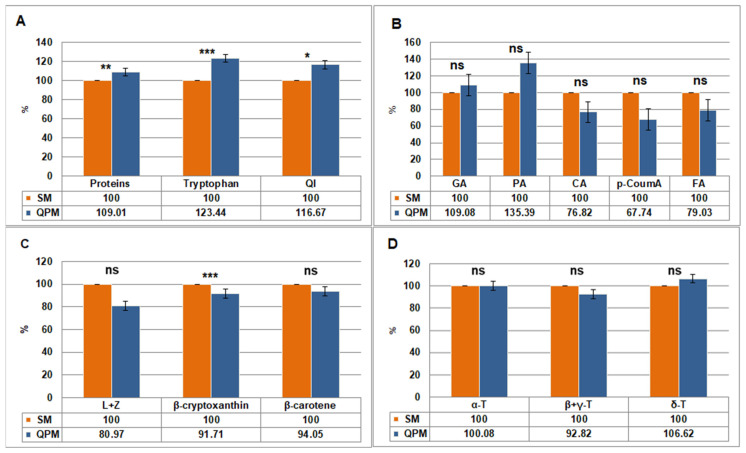
Differences (%) in the average content of biochemical compounds between standard maize (SM) (taken as 100%) and quality protein maize (QPM) lines: proteins, tryptophan, and QI (**A**), free phenolic acids (**B**), carotenoids (**C**), and tocopherols (**D**). GA—gallic acid, PA—protocatechuic acid, CA—caffeic acid, p-Coum—p-coumaric acid, FA—ferulic acid; L + Z—lutein + zeaxhantine. Student’s *t*-test was used to determine the significance of differences between the results obtained between SM and QPM lines: *—statistically significant at *p* < 0.05; **—statistically significant at *p* < 0.01; ***—statistically significant at *p* < 0.001; ns—not significant. Error bars represent standard errors.

Average protein and tryptophan contents, as well as QI, were higher in QPM compared to SM lines (Table 1). The percentage of their increase in QPM (SM taken as 100%) is presented in Figure 1A. Compared to their SM counterparts, all three quality traits were significantly higher (*p* < 0.05) in most QPM lines (Table S1). The exceptions included L1QPM2, which showed no change in protein content, and L6QPM, which exhibited an insignificant increase in tryptophan. Among all lines, L7QPM had the highest tryptophan content, followed by all three QPM versions of the L1 SM inbred line. In lines L4QPM and L5QPM, tryptophan content was below the QPM threshold (0.075), although still significantly higher compared to their SM counterparts. Quality index was significantly increased in all QPM lines except L2QPM; however, only L7QPM exceeded the QPM threshold of 0.80.

#### 2.1.2. Free Phenolic Acid Content

On average, QPM lines showed increased GA and PA contents, and decreased CA, p-CoumA, and FA contents compared to SM lines (Table 1). However, none of these differences were statistically significant. The percentage of phenolic acids contents changes in QPM (SM taken as 100%) is presented in Figure 1B. Some consistent patterns between SM and their QPM counterparts could be noted (Table S1). Thus, GA was statistically higher in all QPM versions except L1QPM1 and L1QPM2, where it was on par with L1. In contrast, p-CoumA acid was significantly lower in all QPM counterparts. No clear pattern was observed for the remaining three phenolic acids.

#### 2.1.3. Carotenoid Content

Average contents of lutein + zeaxanthin, β-cryptoxanthin, and β- were higher in SM compared to QPM lines (Table 1). However, Student’s *t*-test revealed a statistically significant difference between SM and QPM only for β-cryptoxanthin content (*p* < 0.001). The percentage of carotenoids contents changes in QPM (SM taken as 100%) is presented in Figure 1C. Carotenoids contents of individual SM lines and their QPM counterparts are given in Table S1. They were undetectable in L7 and L7 QPM because these are white kernel maize lines. Overall, contents of the carotenoids in QPM lines were either statistically lower or on par with their SM counterparts. The only exceptions were L + Z and β-carotene in L6QPM, which were statistically higher compared to L6.

#### 2.1.4. Tocopherol Content

Although the average β + γ-tocopherols content was higher in SM lines and δ-tocopherol in QPM lines, the differences were not statistically significant (Table 1). The average α-tocopherol content was the same in both groups. The percentage of tocopherols contents differences between QPM and SM (taken as 100%) lines is presented in Figure 1D. Considering individual SM lines and their QPM counterparts, no consistent pattern in tocopherol content was observed between them (Table S1). In most cases, either a statistically significant decrease or no statistically significant change was noted.

QI—quality index, GA—gallic acid, PA—protocatechuic acid, CA—caffeic acid, p-Coum—p-coumaric acid, FA—ferulic acid; L + Z—lutein + zeaxhantine; ns—not significant.

### 2.2. Germination Parameters

#### 2.2.1. Seed Germination

**Figure 2:**
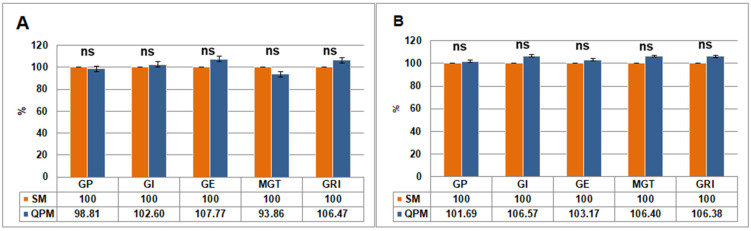
Difference (%) in average seed germination parameters between standard maize (SM) (taken as 100%) and quality protein maize (QPM) lines under optimal temperature (25°/22 °C) (**A**) and cold stress (**B**). Student’s *t*-test was used to determine the significance of differences between the results obtained between SM and QPM lines under the same conditions: ns—not significant. Error bars represent standard errors.

Average germination parameters values for SM and QPM under optimal and cold stress conditions are presented in Table 2. Student’s *t*-test indicated significant differences between optimal and stress conditions only for GE within each group, while all other parameters showed no significant differences. Difference (%) in average germination parameters between SM (taken as 100%) and QPM under optimal temperature and under low temperature stress are presented in Figure 2. Under optimal conditions, QPM lines generally performed better than SM lines in most germination parameters, with the exception of GP (Figure 2A). Under the cold stress, MGT was the only parameter favoring SM (Figure 2B). However, none of the differences between SM and QPM lines under either condition were statistically significant for any germination parameter.

Fisher’s LSD_0.05_ analysis results for germination parameters are presented in Table S2. Under cold stress, no significant differences in GP were detected between most lines, except for L2QPM, which differed significantly from several lines but not from its SM counterpart. On the other hand, GI was significantly higher in all QPM counterparts compared to their SM originals, with the exception of L2QPM and L7QP, which showed either a significant decrease or no difference relative to L2 and L7, respectively. Differences in GE between SM and their corresponding QPM lines were not statistically insignificant, except for L5 and L5QPM. Considering MGT, most QPM lines showed either no significant differences or significant improvement in comparison with SM originals. Finally, GRI was significantly higher in four QPM lines, significantly lower in one, and unchanged in four relative to their SM originals. Among all lines, L3QPM consistently ranked highest across all five germination parameters under cold stress.

**Figure 3:**
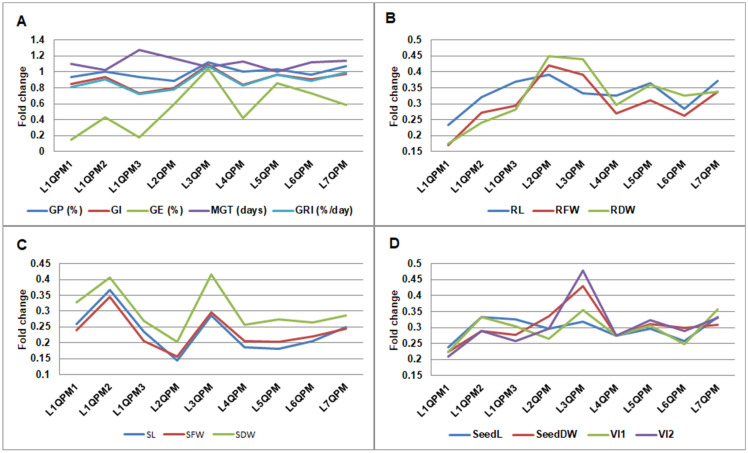
Fold change in germination parameters (**A**), root traits (**B**), shoot traits (**C**), and seedling traits and vigor indices (**D**), caused by cold stress treatment of quality protein maize (QPM) lines.

The fold decrease in germination parameters for QPM lines under cold stress is presented in Figure 3A. Germination energy was the most severely affected parameter in all analyzed lines, with the exception of L3QPM. This line showed best performance under cold stress, maintaining germination at the same level as under optimal conditions.

#### 2.2.2. Seedling Morphological and Physiological Traits

**Figure 4:**
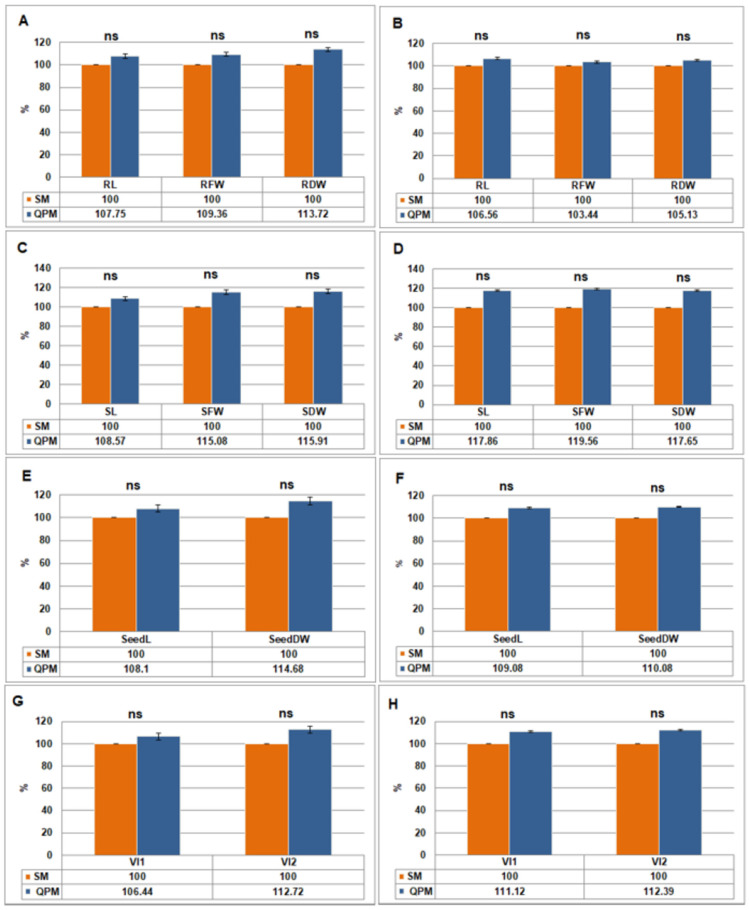
Difference (%) in average morphological and physiological parameters between standard maize (SM) (taken as 100%) and quality protein maize (QPM) lines under optimal temperature (25°/22 °C) and cold stress (13°/6 °C). Root traits under optimal temperature (**A**) and under cold stress (**B**). Shoot traits under optimal temperature (**C**) and under cold stress (**D**). Seedling traits under optimal temperature (**E**) and under cold stress (**F**). Vigor indices under optimal temperature (**G**) and under cold stress (**H**). Student’s *t*-test was used to determine the significance of differences between the results obtained between SM and QPM lines under the same conditions: ns—not significant. Error bars represent standard errors.

Average values for root and shoot morphological and physiological trait, as well as of SeedL, SeedDW, VI1 and VI2, are shown in Table 2. Statistically significant differences (*p* < 0.001) between optimal and stress conditions were found for all traits in both SM and QPM lines. On the other hand, although average values under both optimal and stress conditions were higher for QPM lines, the differences were not statistically significant (Figure 4).

Fisher’s LSD_0.05_ analysis results are given in Table S3. For root traits under cold stress, all QPM lines showed either significantly higher or insignificant difference compared to their SM originals. The only exception was L2QPM, which showed significantly lower root traits. For shoot traits under cold stress, differences between SM and their QPM counterparts were for the most part not significant. Similar results to root traits were observed for VI1 and VI2 under cold stress. Among all analyzed lines, L3QPM ranked as the best or among the best across all morphological and physiological traits, while L7QPM performed the best for RL, all three shoot traits, and VI1.

The fold decrease in morphological and physiological traits for QPM lines under cold stress is presented in Figure 3. Overall, shoot traits were more severely affected than root traits, seedling traits or vigor indices. Across all three groups of traits, L3QPM was the QPM line least affected by cold stress.

### 2.3. Pearson’s Correlations Analysis

#### 2.3.1. Germination Parameters and Biochemical Traits

Under optimal conditions, significant positive correlations were observed between three phenolic acids (GA, pCoumA, FA) and GI, GE, and GRI (Table S4a). Under cold stress, GA and pCoumA maintained positive correlations with GI and GE, while PA showed significant negative correlations with GP and GI. Significant negative correlations were also found between protein content and GI and GE, while positive correlations were found with MGT. No significant correlations were found between carotenoids or tocopherols and any of the germination parameters under either condition.

#### 2.3.2. Morphological/Physiological Traits and Biochemical Traits

Phenolic acids also showed significant correlations with morphological and physiological traits (Table S4b), with some differences between optimal and stress conditions. Thus, GA was significantly correlated with all three shoot traits under optimal temperature, but with SDW, SeedDW, and VI2 under the stress. Also, p-CoumA was correlated with SL under optimal conditions and additionally with RFW and RDW under cold stress. On the other hand, significant negative correlations were found for PA with SFW and SDW under both temperature conditions. Tryptophan was significantly positively correlated with RL under optimal conditions, whereas under the stress, carotenoids (L + Z and β-cryptoxhantin) were significantly negatively correlated with RL, SeedL, and VI1. Tocopherols showed no significant correlations with any morphological or physiological traits under either condition.

#### 2.3.3. Morphological/Physiological Traits and Germination Parameters

Under optimal conditions, all shoot traits and SeedL, were significantly correlated with all germination parameters except GP (Table S4c). SeedDW was significantly correlated with MGT and GRI, VI1 with GI, GE, and GRI, and VI2 with GRI. No significant correlations were found between root traits and germination parameters. However, under cold stress, all morphological and physiological traits were significantly correlated with GI and GRI. Similarly, all traits except SL were correlated with GE. Additionally, all shoot traits were significantly correlated with GP, while RFW, RDW, SeedDW, and VI2 were significantly correlated with MGT.

## 3. Discussion

The urgent need to develop maize hybrids and varieties with greater adaptability to climate change has been widely recognized. Although the global average temperature has increased by 1.1 °C, recent estimates indicate that the Republic of Serbia is experiencing a more intense and rapid warming trend—an average temperature increase of 1.8 °C, with summer temperatures rising by as much as 2.6 °C [21]. These extreme climatic and weather conditions are already contributing to significant reductions in crop production. Consequently, effective mitigation measures are urgently required. These include adjusting sowing dates, optimizing plant densities based on specific hybrids and regional conditions, and improving overall crop management practices [22]. An additional strategy for these measures could involve developing maize with high nutritive value to ensure not only the quantity but also grain quality and to consequently support the dietary needs of a growing human population. In this context, the potential of ZPQPM lines for the development of climate-resilient maize with high nutritive value is explored further in the following sections.

### 3.1. Seed Nutritive Composition

Quality protein maize is nutritionally superior compared with standard maize due to its elevated levels of the essential amino acids lysine and tryptophan. The QPM counterparts of ZP commercial inbred lines demonstrated increased tryptophan content. While lysine content was not directly measured, its improvement can be reasonably assumed, given the strong correlation between lysine and tryptophan levels (r > 0.90) [23]. The same authors also defined benchmark values for QPM: 0.075% tryptophan and 0.80% QI in the sample. All analyzed QPM lines had acceptable tryptophan content. Even though L4QPM and L5QPM lines had somewhat lower tryptophan content, it was still significantly higher than in their SM originals. Moreover, it can be expected that protein and tryptophan contents in these lines could increase when grown in more favorable environmental conditions, as previous studies have shown that drought stress can enhance their accumulation [24,25].

Quality index was low for most of the analyzed converted lines. Only line L7QPM exceeded the defined threshold of 0.80%, fulfilling both required benchmarks. Although all other QPM lines, except L2QPM, showed a statistically significant increase in QI, indicating an improved tryptophan-to-protein ratio and thus increased tryptophan availability, their QI values remained within the range characteristic of standard maize [26]. Overall, most of the converted analyzed lines denoted as QPM can be considered nutritionally superior to their SM originals, primarily due to significantly higher contents of tryptophan and lysine, as well as improved protein quality. However, to fully evaluate their nutritional value, further analysis of other essential amino acids is required, as it was shown that histidine, threonine, and methionine can also be increased in QPM [27,28]. Moreover, leucine is often reduced in QPM [27,28,29,30], resulting in a more balanced leucine-to-isoleucine ratio, which further enhances overall protein quality.

The SM and QPM lines were also analyzed for their carotenoid, tocopherol, and phenolic acid contents. These secondary metabolites were chosen for evaluation due to their pronounced health benefits. In addition to their specific roles as health promoting compounds, they are all strong antioxidants, contributing to reduced risks of certain types of cancer and cardiovascular diseases [31,32,33]. The carotenoids and tocopherols contents in this study were consistent with the findings of Calugar [34] and Šarčević [35], respectively. In contrast, the contents of free phenolic acids were higher than those reported by Hadinezhad [36], who noted that developmental stage, environmental conditions, and genotype can all influence their content.

The average contents of the analyzed metabolites differed between SM and QPM lines, though not significantly; this was not the case for β-cryptoxhantine however, which was significantly higher in SM. Since the genes and biochemical pathways involved in tryptophan and carotenoid biosynthesis are distinct [37,38], this difference may be attributed to unintended genetic recombination affecting non-target genes during the MAS conversion of SM to QPM. At the level of individual SM lines and their QPM counterparts, no consistent patterns were observed. Carotenoid and tocopherol contents in QPM were either statistically lower or unchanged. Similar variability was found in phenolic acid contents, which were randomly increased, decreased or unchanged—except for p-coumaric acid. This phenolic acid was significantly lower in all QPM lines. This is in line with the findings of Tarasevičiene [39], who reported that increased tryptophan content may lead to a reduction in p-coumaric acid levels.

Among the individual QPM lines, three high tryptophan (over 0.075%) lines with significantly increased QI (but not below 0.60%) also showed significantly elevated contents of some tocopherols and/or phenolic acids. Line L1QPM2 was improved for both α-T and δ-T, as well as for CA contents. Line L3QPM showed improvements in β + γ − T, GA, and CA. The line with the highest tryptophan content and QI, L7QPM, was enriched for one phenolic acid—GA. These findings imply enhanced health benefits due to the distinct roles of these compounds. Tryptophan is essential for numerous vital metabolic processes [40], α-T is a potent antioxidant that protects cell membranes [41], CA demonstrated beneficial effects in the prevention and management of various cancers, diabetes, and neurodegenerative disorders [42], while GA participates in multiple biological mechanisms associated with both pro- and anti-inflammatory pathways, and exhibits cardio- and gastro-protective properties [43].

### 3.2. Cold Stress Tolerance

Cold tolerance of SM lines and their QPM counterparts during germination was assessed to evaluate the potential of using nutritionally improved genotypes in breeding programs aimed at developing hybrids suitable for early sowing. To ensure accurate results, multiple germination parameters were calculated. It is well established that final germination percentage alone is insufficient for comparing datasets, as the time required for germination is also crucial for obtaining reliable results [44]. The same authors highlighted GI as the most comprehensive parameter, as it integrates both percentage and speed (spread, duration and time at which majority/minority of seeds germinate). The analysis of SM and QPM lines revealed that both groups had the same level of tolerance during germination, as no significant differences were found between them under stress conditions for any of the analyzed parameters.

Interestingly, no statistically significant difference in germination parameters was found between optimal and stress conditions for either type of lines, except for GE. This finding suggests that cold stress has the greatest impact on germination energy. It is well known that plants undergo a series of physiological and morphological changes under low temperature stress, such as ROS accumulation, metabolic disorders, delayed germination, reduced germination rate, and inhibited seedling growth [45]. These changes result in low germination energy, which reflects the rate and uniformity of germination within a defined, short period. The lack of significant changes in the other four parameters could be explained by high seed viability and the fact that these parameters assess overall germination success, even if delayed.

Significant correlations were found between some biochemical compounds and germination parameters. Four phenolic acids were correlated with two to four germination parameters under optimal (FA), stress (PA) or both optimal and stress (GA and p-CoumA) conditions. This is not surprising, as it is well known that free phenolic acids have important and specific roles during seed germination, including germination under temperature stress, due to their potent ability to scavenge ROS, form complexes with metals, and raise the activity of oxidative enzymes [46,47]. Besides phenolic acids, proteins also exhibited strong correlations with GI, GE, and MGT, but only under the stress conditions. This could be explained by the fact that seed proteins play a crucial role in both the inhibition and modulation of the germination process under cold stress through different mechanisms, affecting germination rates and seedling development under low temperatures [48,49].

Analyses of roots, shoots, seedling traits, and vigor indices revealed no statistically significant differences between SM and QPM under either optimal or cold stress conditions. However, under the stress, highly significant differences were noted for all analyzed traits for both SM and QPM lines. At the seedling stage, low-temperature stress has the strongest effects on the degree of germination, seedling rate, and seedling potential, significantly impacting stem and root development [50]. These are primarily due to the molecular changes, including disruptions in membrane structure [51], alterations in antioxidant enzymes activity [52], and hormone levels [53]. Both SM and QPM lines were severely affected by cold stress, with the reduction in root and shoot traits up to sixfold (e.g., RL in L1QPM1) or even sevenfold (e.g., SL in L2QPM). A greater decrease under low temperature was noted for shoot traits, being 10.09% and 6.68% on average for SM and QPM, respectively. A somewhat lower reduction in high tryptophan QPM lines could be explained by the findings of Ren [54], who showed that melatonin, derived from tryptophan, is essential for maintaining vital processes in cells, such as cell membrane stability, osmotic balance, antioxidative defense, and photosynthetic activity. The primary reason for stronger impact of cold stress on shoot traits could be attributed to significant photosynthetic inhibition, as photosynthesis is the most stress-sensitive physiological process [55]. Other contributing factors include ROS accumulation, reduced transpiration, antioxidant enzyme activity, and chlorophyll content, as well as structural damage [56]. Furthermore, cold stress at early developmental stages leads to abnormalities in chloroplast structure or delays full development of photosynthetic apparatus [57].

Considering correlations with analyzed biochemical compounds, shoot and/or root traits were significantly correlated with tryptophan, three phenolic acids (GA, PA, p-CouM), L + Z, and β-cryptoxhantin. GA and PA were significantly correlated with shoot traits under both optimal and cold stress conditions. On the other hand, p-CouA was significantly correlated with SL under both conditions, as well as with RFW and RDW under stress. High correlations with shoot traits could be explained by phenolic acid’s role in photoprotection (UV absorption, ROS scavenging, NPQ) within the photosynthetic tissues of the shoots [58]. Metabolic processes in roots do not depend on light in the same way as in shoots, and as a result the need for light-protective phenolics is reduced. However, correlations between p-coumaric acid and RFW and RDW suggest involvement in the broader metabolic reprogramming that occurs in plants to adapt cold conditions. Carotenoids are precursors of plant hormones strigolactones (SLs) and abscisic acid (ABA), as well as multiple bioactive molecules, which are regulators of root development and architecture [59]. The negative correlations found between root length and L + Z/β-cryptoxanthin could be the consequence of the high complexity of stress regulation mechanism, including dual functions of phytohormones. For instance, ABA can promote root growth under mild stress, but under severe stress such as the cold stress applied in this research, it may also inhibit root elongation and development [60].

Seed vigor is a complex agronomic trait, defined as the capacity of seeds to germinate and emerge quickly, particularly under stressful conditions. It plays a crucial role in determining the duration and success of seedling establishment, which is vital for achieving high yields [61]. Manjeet [62] reported that the combined results of correlation coefficient and path coefficient analysis identified VI1, shoot length, and seedling length as major components of standard germination in sesame. Similarly, association mapping of rice seedling traits revealed QTLs for germination percentage, VI1, VI2, root growth rate, and root-to-shoot ratio, all of which could be used in breeding for improved seed vigor [63]. Additionally, Zhang [64] successfully used GP, GE, and VI1 for comprehensive and rapid evaluation of wheat seed vigor through hyperspectral imaging. Similarly, in our work, GI, GE, and GRI were significantly correlated with both VI1 and VI2 under both optimal and stress conditions. Under cold stress, correlation strength (R scores) and statistical significance between VI2 and these three germination parameters were increased. Furthermore, MGT was significantly correlated with VI2 under the stress, while no change was observed for VI1. These results indicate that cold stress had a more profound effect on biomass accumulation and robustness of the seedlings compared to the speed and uniformity of germination or the initial growth rate.

The previous field assessments of SM lines, as observed by breeders, were not based on an experimental design or early sowing. A comparison between these field observations and results obtained under controlled conditions, which also refer to different developmental stages, indicates varying levels of tolerance for each line. For example, L1 was considered tolerant, while L2 was considered susceptible by the breeders. However, under cold stress treatment during germination, L1 exhibited the lowest GE and VI1, while L2 showed one of the highest values for both parameters. This implies the necessity of more comprehensive research, including field experiments. It has been shown that early sowing success depends on various factors such as soil texture and pH, precipitation, and solar radiation [17,19]. Multi-year and multi-site trials are necessary to establish an accurate and reliable planting calendar. Nevertheless, the results presented in this paper are beneficial as the first step, as they identified genotypes for further research.

## 4. Materials and Methods

### 4.1. Plant Material

A total of 16 inbred lines developed at MRIZP were chosen for the experiment. Seven ZP commercial lines were original standard maize (SM) dent lines and nine were their QPM counterparts. QPM was developed through marker-assisted selection (MAS) with 3–4 backcrosses, in which an adapted QPM ZP line [8] was used as a donor line. The list of the lines, their type (SM or QPM), and cold tolerance assessment of SM lines based on previous breeders’ experience in field after emergence, as well as FAO and heterotic groups, are presented in Table 3.

### 4.2. Seed Biochemical Analyses

#### 4.2.1. Protein and Tryptophan Content Determination

Each inbred line was represented by 60 kernels, divided into two samples consisting of 30 kernels each. Kernels were dried in a controlled oven (COLO LabExperts, Novo mesto, Slovenia) at 65 °C overnight (16–18 h) and milled in a lab mill (Perten 120, Perten Instruments, Hägersten, Sweden). The flour was defatted by hexane treatment for 4 h in Soxhlet extractor (INKOLAB, Zagreb, Croatia).

Tryptophan content (TC) was determined using the colorimetric method given in Twumasi-Afriyie [23]. The color was developed in the reaction of flour hydrolysate (obtained by overnight digestion with papain solution at 65 °C) with glyoxylic acid and ferric chloride dissolved in sulfuric acid. After incubation at 65 °C for 30 min, absorbance was read on spectrophotometer (Shimadzu UV-1601) at 560 nm. Tryptophan content was calculated using a standard calibration curve, developed with known amounts of tryptophan, ranging from 0 to 30 μg mL^−1^.

The protein content (PC) was determined by the standard Kjeldahl method using FOSS Kjeltec^TM^ 8400 (FOSS, Hillerød, Sweden), based on nitrogen (N) determination as explained in Vivek [65]. The protein was estimated from the N value as follows: $PC\left(\%\right)=N\left(\%\right)\times 6.25(\mathrm{conversion}\mathrm{factor})$.

Quality index (QI), defined as tryptophan to protein ratio in the sample, was calculated as QI=100×(tryptophancontentinthesample/proteincontentinthesample).

#### 4.2.2. Tocopherols, Carotenoids, and Free Phenolic Acids Determination

Approximately 0.5 g, 0.15 g, and 0.2 g of maize flour (particle size < 500 μm) were used to determine the content of free phenolic acids, carotenoids, and tocopherols, respectively. Extraction of free phenolic acids (gallic acid (GA), protocatechuic acid (PA), caffeic acid (CA), p-coumaric acid (p-CoumA), and ferulic acid (FA)), carotenoids (lutein + zeaxanthin (L + Z), β-cryptoxanthin, and β-carotene) and tocopherols (α − T, β + γ – T, and δ − T) was performed according to the method of Vukadinović [66], with modifications in the weight to volume ratio (*w*/*v*). For free phenolic acids, carotenoids, and tocopherols, the *w*/*v* ratios were 1:20, 1:25, and 1:20, respectively. The chromatographic separation of free phenolic acids was performed using the Acclaim Polar Advantage II, C18 (150 × 4.6 mm, 3 μm) analytical column, while the Hypersil GOLD C18 (150 × 4.6 mm, 3 μm) and Hypersil GOLD aQ C18 (150 × 3 mm, 3 μm) analytical columns were used for the separation of carotenoids and tocopherols. The injection volume, column temperature, Chromeleon software package (version 7.2) for instrument control and data analysis, and the mobile phase used for the separation of free phenolic acids, carotenoids, and tocopherols were set as described in the studies by Mesarović [67] and Mesarović [68]. The content of the analyzed bioactive compound (secondary metabolites) is expressed as μg per g of dry weight (dw) and reported as the mean value of three independent injections.

### 4.3. Cold Stress Experiment

#### 4.3.1. Experimental Design

The experiment was carried out in three replicates of 30 seeds for each genotype. After the sterilization in 1% sodium hypochlorite (commercial bleach), seeds were placed on, covered with, and rolled in the moist filter paper thoroughly soaked with distilled water, and left in a growth chamber for seven days (light intensity 700 μ mol m^−2^ s^−1^, photoperiod 12 h/12 h, humidity approx. 60%). After the initial 48 h imbibition period under optimal temperature conditions (25°/22 °C day/night) seedlings were exposed to low temperature (13°/6 °C day/night) for five more days. Control samples were grown under optimal conditions for seven days. The number of germinated seeds was scored daily until the 7th day. Out of 30 seedlings from each replicate, 10 were chosen for morphological and physiological traits measurement. The cold stress temperature (13°/6 °C day/night) is the median of maximum and minimum temperatures recorded in the period from the 20th of March to the 10th of April for six years with cold early springs over a 12-year period from 2012 to 2023 (provided by the Republic Hydrometeorological Service of Serbia), representing predictive tendency of temperatures that maize seedlings would be exposed to when sown early (Figure S1).

#### 4.3.2. Germination and Vigor Determination

Germination percentage (GP), germination index (GI), germination energy (GE), mean germination time (MGT), and germination rate index (GRI), as well as vigor indices VI1 and VI2, were calculated according to the formulas given in Kader [44]:

(1) $$GP\left(\%\right)=\frac{\mathrm{number}\mathrm{of}\mathrm{germinated}\mathrm{seeds}}{\mathrm{total}\mathrm{number}\mathrm{of}\mathrm{seeds}}\times 100$$

(2) GI=(7×n1)+(6×n2)+(5×n3)+(4×n4)+(3×n5)+(2×n6)+(1×n7),

where n1, n2…n7 are the number of germinated seeds on day 1, 2, …, 7, and numbers 7, 6, …, 1 are weights given to the number of germinated seeds on the first, second, and subsequent days.

(3) $$GE\left(\%\right)=\frac{\mathrm{number}\mathrm{of}\mathrm{germinated}\mathrm{seeds}\mathrm{on}\mathrm{day}3}{\mathrm{total}\mathrm{number}\mathrm{of}\mathrm{seeds}}\times 100$$

(4) $$MGT\left(days\right)=\frac{\sum f\times x}{\sum f},$$

where f is number of germinated seeds on day x.

(5) $$GRI\left(\%/day\right)=\frac{G1}{1}+\frac{G2}{2}+\frac{G3}{3}+\frac{G4}{4}+\frac{G5}{5}+\frac{G6}{6}+\frac{G7}{7},$$

where G1, G2, …, G7 are per cents of germinated seeds on day 1, 2, …, 7.

Vigor indices VI1 and VI2 were calculated according to Abdul-Baki and Anderson [69]:


(6) VI1=GP×seedlinglength(shootlength+rootlength)

(7) VI2=GP×seedlingdryweight(shootdryweight+rootdryweight)

#### 4.3.3. Seedling Morphological and Physiological Traits

Seedling traits measured include root length (RL) given in cm, as well as root fresh weight (RFW) and dry weight (RDW) given in grams. After fresh weight measurement, samples were dried in an oven at 110 °C for 24 h and measured for dry weight. The same measurements were performed for shoots—length (SL), fresh weight (SFW), and dry weight (SDW). Additionally, seedling length (RL + SL) and dry weight (RDW + SDW) were calculated for VI1 and VI2 determination, respectively.

### 4.4. Statistical Analysis

For biochemical traits, one factorial analysis of variance (ANOVA) according to RCBD was performed, while for root/shoot traits and germination parameters, two factorial ANOVA was used. Fisher’s least significant difference test (LSD) at 0.05 probability level was used to estimate the significances of differences between the observed means. These statistical analyses were performed in MSTAT-C software [70]. Student’s *t*-test (Microsoft Excel) was used to determine the significance of differences between the results obtained under optimal temperature conditions and cold stress treatment, as well as between SM and QPM lines under the same conditions. Pearson’s correlation coefficients (Microsoft Excel 2010) were calculated between the estimated biochemical and germination parameters, between biochemical parameters and root/shoot traits and vigor indices, as well as between germination parameters and root/shoot traits and vigor indices. Statistical significance of the correlations was determined using a Table of Critical Values [71].

## 5. Conclusions

Cold stress had significant negative impact on GE, but other germination parameters, including GP, GI, MGT, and GRI, remained unchanged. This suggests that, although germination was delayed, it was successful overall for most of the analyzed lines. While all morphological and physiological traits were strongly impacted by the stress, shoot traits displayed the highest susceptibility, probably due to the inhibition of photosynthesis. Furthermore, it was observed that some phenolic acids and carotenoids contribute to cold tolerance during germination and early development. No significant differences were found between SM and QPM lines under either optimal or stress conditions, indicating that the tolerance was maintained during the MAS conversion. In terms of nutritional value, most QPM lines showed enhanced protein and tryptophan content, as well as improvements in one or more secondary metabolites. Three QPM lines with improved nutritional quality—L1QPM2, L3QPM, and L7QPM—were also among the lines most tolerant to cold stress. Line L3QPM excelled among all lines, exhibiting unchanged GE and the lowest fold change in both VI1 and VI2 under stress conditions. It can be said that these three QPM lines are promising candidates for breeding high nutritive maize tolerant to cold stress and are suitable for early sowing.

## Figures and Tables

**Table 1:** Summary of biochemical analyses of standard maize (SM) lines and their quality protein maize (QPM) counterparts.

Group of Biochemical Compounds	Biochemical Compounds	SM		QPM		Difference (QPM-SM)	Level of Difference Significance
		Average	SD	Average	SD		
Proteins, amino acids (%)	Total proteins	11.76	0.06	12.82	0.06	1.06	0.01
	Tryptophan	0.064	0.001	0.079	0.003	0.015	0.001
	QI	0.54	0.07	0.63	0.08	0.09	0.05
Phenolic acids (μg/g dm)	GA	22.36	0.58	24.39	0.85	2.03	ns
	PA	8.28	0.18	11.21	0.14	2.93	ns
	CA	8.80	0.13	6.76	0.09	−2.04	ns
	p-CoumA	3.10	0.05	2.10	0.03	−1.00	ns
	FA	4.72	0.06	3.73	0.04	−0.99	ns
Carotenoids (μg/g dm)	L + Z	24.65	1.28	19.96	1.39	−4.69	ns
	β-cryptoxanthin	5.31	0.23	4.87	0.26	−0.44	0.001
	β-carotene	4.87	0.09	4.58	0.14	−0.29	ns
Tocopherols (μg/g dm)	A − T	12.59	0.27	12.60	0.27	0.01	ns
	β + γ − T	43.86	1.16	40.71	1.08	−3.15	ns
	Δ − T	1.51	0.04	1.61	0.05	0.10	ns

**Table 2:** Average values of germination parameters, root and shoot traits, seedling traits and vigor indices of standard maize (SM) and quality protein maize (QPM) lines under control (optimal temperature) and treatment (cold stress) conditions.

Group of Traits	Traits	Average				*t*-Test (C/T)	
		SM		QPM			
		Control	Treatment	Control	Treatment	SM	QPM
Germination parameters	GP (%)	97.46	93.97	96.30	95.56	ns	ns
	GI	415.86	360.00	426.67	383.67	ns	ns
	GE (%)	76.98	46.67	82.96	48.15	*	***
	MGT (days)	3.26	3.20	3.06	3.41	ns	ns
	GRI (%/day)	31.01	27.60	33.01	29.36	ns	ns
Root traits	RL (cm)	14.58	4.88	15.71	5.20	***	***
	RFW (g)	0.2649	0.0843	0.2897	0.0872	***	***
	RDW (g)	0.0226	0.0078	0.0257	0.0082	***	***
Shoot traits	SL (cm)	6.77	1.40	7.35	1.65	***	***
	SFW (g)	0.2222	0.0496	0.2557	0.0593	***	***
	SDW (g)	0.0176	0.0051	0.0204	0.0060	***	***
Seedling traits	SeedL (cm)	21.34	6.28	23.07	6.85	***	***
	SeedDW (g)	0.0402	0.0129	0.0461	0.0142	***	***
Vigor indices	VI1	2075.91	589.92	2238.47	669.48	***	***
	VI2	3.91	1.21	4.34	1.43	***	***

*—statistically significant at *p* < 0.05; ***—statistically significant at *p* < 0.001; ns—not significant.

**Table 3:** The list of standard lines (SM) and their quality protein maize (QPM) counterparts used for cold stress tolerance assessment.

#	Inbred Line	Type	* Cold Tolerance	FAO Group	Heterotic Group
1	L1	SM	moderately tolerant	FAO 550	Lancaster
2	L1QPM1	QPM	-		
3	L1QPM2	QPM	-		
4	L1QPM3	QPM	-		
5	L2	SM	susceptible	FAO 350–400	Lancaster
6	L2QPM	QPM	-		
7	L3	SM	tolerant	FAO 300	BSSS/ID
8	L3QPM	QPM	-		
9	L4	SM	tolerant	FAO 300	BSSS/ID
10	L4QPM	QPM	-		
11	L5	SM	tolerant	FAO 450–500	BSSS/ID
12	L5QPM	QPM	-		
13	L6	original	moderately tolerant	FAO 300	BSSS/ID
14	L6QPM	QPM	-		
15	L7	original	moderately tolerant	FAO 600	BSSS
16	L7QPM	QPM	-		

* arbitrarily estimated cold tolerance in the field by breeders after emergence; ”-”: no data.

## Data Availability

Data are contained within the article.

## Supplementary Materials

The following supporting information can be downloaded at: https://www.mdpi.com/article/10.3390/plants14162540/s1, Figure S1: Median maximum and minimum temperatures recorded in the period from the 20th of March to the 10th of April over a 12-year period from 2012 to 2023; Table S1: Biochemical analyses of SM and QPM lines and Fishers LSD; Table S2: Germination parameters under control (optimal temperature) and treatment (cold stress) conditions and Fishers LSD; Table S3: Results of SM and QPM morphological and physiological trait analyses with Fishers LSD; Table S4a: Pearsons correlation coefficients between germination parameters and biochemical compounds; Table S4b: Pearsons correlation coefficients between morphological and physiological traits and biochemical compounds; Table S4c: Pearsons correlation coefficients between morphological and physiological traits and germination parameters.
