# Supplementary material for: Early Sowing Approach for Developing Climate Resilient Maize: Cold Stress Impact on Germination of Adapted Inbred Lines with High Nutritive Value
Source: Plants (Basel). 2025 Aug 15;14(16):2540. doi: 10.3390/plants14162540 (PMC12389089; doi:10.3390/plants14162540)
Supplement: Supplementary file 1 [file plants-14-02540-s001.zip › plants-3789613-supplementary/Figure S1.pdf]

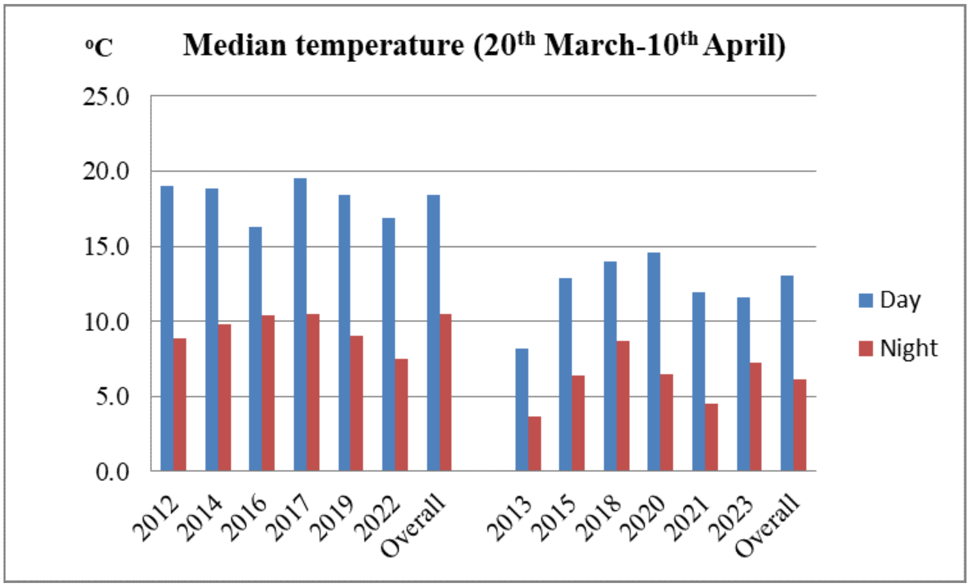

Figure S1. Median maximum and minimum temperatures recorded in the period from the 20th of March to the 10th of April over a 12-year period from 2012 to 2023.

Median temperatures					
Maximum t>15˚ C			Maximum t<15˚ C		
Year	t_{max}	t_{min}	Year	t_{max}	t_{min}
2012	19.0	8.9	2013	8.2	3.7
2014	18.8	9.8	2015	12.9	6.4
2016	16.3	10.4	2018	14.0	8.7
2017	19.5	10.5	2020	14.6	6.5
2019	18.4	9.0	2021	11.9	4.5
2022	16.9	7.5	2023	11.6	7.2
Overall	18.4	10.5	Overall	13.0	6.1

Median maximum and minimum temperatures recorded in the period from the 20th of March to the 10th of April over a 12-year period from 2012 to 2023 (provided by the Republic Hydrometeorological Service of Serbia) are presented.

Years considered to be with cold stress are those with median maximum temperatures below 15˚ C - 2013, 2015, 2018, 2020, 2021 and 2023. The cold stress temperature (13˚/6˚ C day/night) was defined as the overall median maximum and minimum temperatures of these years.
