# Supplementary material for: Early Sowing Approach for Developing Climate Resilient Maize: Cold Stress Impact on Germination of Adapted Inbred Lines with High Nutritive Value
Source: Plants (Basel). 2025 Aug 15;14(16):2540. doi: 10.3390/plants14162540 (PMC12389089; doi:10.3390/plants14162540)
Supplement: Supplementary file 1 [file plants-14-02540-s001.zip › plants-3789613-supplementary/Table S1 revised.pdf]

Table S1. Biochemical analyses of SM and QPM lines and Fishers LSD.

#	Genotype	Proteins (%)	Tryptophan (%)	QI	Free phenolic acids (µg/g dm)					Carotenoids (µg/g dm)			Tocopherols (µg/g dm)		
					GA	PA	CA	p – CoumA	FA	L+Z	β-cryptoxanthin	β-carotene	α-T	β+γ-T	δ-T
1	L1	13.76c	0.073d	0.53g	12.14i,j	22.89b	3.91i	1.20i	3.61g	34.27a	5.94a	5.04b	12.87e,f	23.15h,i	1.15g
2	L1 QPM 1	14.73a	0.086b	0.58d,e	11.78i,j	19.28c	4.03i	1.09j	2.48j	24.62c	4.54c	4.48c	15.60d	29.63g	1.83e
3	L1 QPM 2	13.74c	0.084b	0.61c	11.03j	19.12c	5.60g	1.01j	2.29k	17.03e	4.13c,d	4.20e	14.95d	25.63h	1.66f
4	L1 QPM 3	14.23b	0.080b,c	0.56e,f	17.15h	17.20d	5.53g	1.00j	2.23k	14.77e	3.94d	4.12e	11.49g	24.41h	1.59f
5	L2	12.40f	0.069e	0.56e,f	23.29f	16.33e	3.70i,j	4.48a	3.12h	21.09d	4.67b,c	4.34c,d	13.25e	15.45j	0.37i
6	L2 QPM	13.60d	0.077c,d	0.57e,f	26.00e	27.29a	1.87k	3.02d	3.99f	17.06e	4.34c,d	4.27c,d	12.70e,f	11.44k	0.33i
7	L3	10.33l	0.057f	0.55f,g	32.42c	5.57f	12.74d	4.47a	8.36a	20.35d	5.31b	5.16b	3.71j	33.71f	1.22g
8	L3 QPM	11.40i	0.076c,d	0.67b	37.92a	5.65f	16.55b	3.99b	7.19c	22.03d	4.68b,c	4.80b,c	3.67j	63.56c	0.95h
9	L4	11.07j	0.068e	0.61c	13.35i	2.50j	4.43h	2.68e	2.96i	29.65b	5.38b	4.53c	9.49h	75.04b	1.20g
10	L4 QPM	11.41i	0.073d	0.64b	28.73d	3.62h	4.33h	1.92g	3.09h	16.07e	3.99d	4.08e	7.32i	73.86b	1.20g
11	L5	12.44f	0.050g	0.40h	34.97b	3.05h,i	13.39c	3.79c	7.59b	20.17d	5.78a,b	5.05b	19.41b	22.27h,i	1.92d
12	L5 QPM	12.72e	0.070d,e	0.55f,g	36.71a	3.14h,i	7.32f	2.59f	5.87d	15.95e	4.56c	4.68c	22.72a	25.23h	2.80a
13	L6	11.62h	0.070d,e	0.60c,d	23.93f	4.97g	18.01a	3.71c	5.04e	22.38c,d	4.76b,c	5.09b	18.17c	82.64a	2.73b
14	L6 QPM	11.75g	0.075c,d	0.64b	28.78d	3.36h	10.01e	3.05d	4.98e	32.19a,b	5.10b	6.03a	13.82e	62.53c	2.55c
15	L7	10.71k	0.058f	0.54f,g	16.40h	2.67j	5.43g	1.38h	2.35k	nd	nd	nd	11.22g	54.78d	1.94d
16	L7 QPM	11.86g	0.096a	0.81a	21.45g	2.19k	5.61g	1.24i	1.48l	nd	nd	nd	11.08g	50.11e	1.59f
	Average	12.36	0.072	0.59	23.5	9.93	7.65	2.54	4.16	21.97	4.79	4.7	12.59	42.09	1.57
	LSD $_{0.05}$	0.12	0.0045	0.03	1.49	0.35	0.22	0.08	0.10	2.5	0.48	0.26	1.09	2.08	0.08
	Average SM	11.76	0.064	0.54	22.36	8.28	8.8	3.1	4.72	24.65	5.31	4.87	12.59	43.86	1.51
	Average QPM	12.82	0.079	0.63	24.39	11.21	6.76	2.1	3.73	19.96	4.87	4.58	12.60	40.71	1.61

QI-quality index, GA-gallic acid, PA-protocatechuic acid, CA-caffeic acid, p-CoumA - p–coumaric acid, FA - ferulic acid, L+Z - lutein + zeaxantin, nd-not detectable. All different letters in the column designate significant differences at 0.05 probability level.
