# Supplementary material for: Early Sowing Approach for Developing Climate Resilient Maize: Cold Stress Impact on Germination of Adapted Inbred Lines with High Nutritive Value
Source: Plants (Basel). 2025 Aug 15;14(16):2540. doi: 10.3390/plants14162540 (PMC12389089; doi:10.3390/plants14162540)
Supplement: Supplementary file 1 [file plants-14-02540-s001.zip › plants-3789613-supplementary/Table S2 revised.pdf]

Table S2. Germination parameters under control (optimal temperature) and treatment (cold stress) conditions and Fishers LSD.

#	Genotype	GP (%)		GI		GE (%)		MGT (days)		GRI (%/day)	
		C	T	C	T	C	T	C	T	C	T
1	L1	96.67a	86.67a,b	349f	253h	48.89f	3.33f	3.99h	3.91e,f,g	25.85f	19.09f
2	L1 QPM 1	100a	93.33a,b	388e	330g,f	66.67d,e,f	10.00e,f	3.69g,h	4.07f,g	28.81e,f	23.33e,f
3	L1 QPM 2	100a	100a	391e	366e	70.00c,d,e,f	30.00d,e,f	3.66g,h	3.73e,f,g	29.15e,f	26.39d,e
4	L1 QPM 3	100a	93.33a,b	426c,d	310g	73.33b,c,d,e	13.33e,f	3.27e,f	4.17g	31.11c,d,e	22.37e,f
5	L2	100a	93.33a,b	450a,b	423b,c	93.33a,b	80a,b	3.00b,c,d,e	2.75a,b	33.89a,b,c,d	33.61b
6	L2 QPM	90a	80b	429c,d	343e,f	90a,b,c	53.33b,c,d	2.70a,b	3.15b,c,d	34.44a,b,c	26.93c,d,e
7	L3	100a	93.33a,b	467a	416b,c,d	100a	73.33a,b,c	2.84b,c,d	3.05b,c,d	35.93a,b	32.69b
8	L3 QPM	86.67a	96.67a	441b,c	480a	86.67a,b,c,d	90.00a	2.35a	2.48a	38.33a	41.11a
9	L4	96.67a	96.67a	396e	326g,f	66.67d,e,f	33.33d,e,f	3.45f,g	3.15b,c,d	29.06e,f	24.35e,f
10	L4 QPM	100a	100a	435b,c	363e	86.67a,b,c,d	36.67d,e	3.17d,e,f	3.57d,e,f	32.06b,c,d,e	26.50d,e
11	L5	93.33a	100a	405e	360e	76.67b,c,d,e	30.00d,e,f	3.18d,e,f	3.40c,d,e	29.72d,e,f	26.56c,d,e
12	L5 QPM	96.67a	100a	454a,b	438b	93.33a,b	80.00a,b	2.78b,c	2.80a,b	36.02a,b	34.81b
13	L6	96.67a	90a,b	437b,c	345e,f	93.33a,b	46.67c,d	2.98b,c,d,e	3.15b,c,d	32.87b,c,d,e	26.02d,e
14	L6 QPM	100a	96.67a	466a	422b,c	100a	73.33a,b,c	2.82b,c,d	3.15b,c,d	36.30a,b	32.15b,c
15	L7	98.89a	97.78a	410d,e	397d	60.00e,f	60.00a,b,c,d	3.39f,g	3.01a,b,c	29.72d,e,f	30.89b,c,d
16	L7 QPM	93.33a	100a	410d,e	401c,d	80a,b,c,d,e	46.67c,d	3.12c,d,e,f	3.54c,d,e,f	30.89c,d,e	30.65b,c,d
	Average	96.81	94.86	421.94	373.31	80.35	47.50	3.15	3.32	32.13	28.59
	$LSD_{0.05}$	30.66	14.6	20.61	24.06	22.76	30.3	0.38	0.56	4.47	5.65
	Average SM	97.46	93.97	415.86	360.00	76.98	46.67	3.26	3.20	31.01	27.60
	Average QPM	96.30	95.56	426.67	383.67	82.96	48.15	3.06	3.41	33.01	29.36

GP-germination percentage, GI-germination index, GE-germination energy, MGT-mean germination time, GRI-germination rate index, C-control, T-treatment. All different letters in the column designate significant differences at 0.05 probability level.
