# Supplementary material for: Early Sowing Approach for Developing Climate Resilient Maize: Cold Stress Impact on Germination of Adapted Inbred Lines with High Nutritive Value
Source: Plants (Basel). 2025 Aug 15;14(16):2540. doi: 10.3390/plants14162540 (PMC12389089; doi:10.3390/plants14162540)
Supplement: Supplementary file 1 [file plants-14-02540-s001.zip › plants-3789613-supplementary/Table S3 revised.pdf]

Table S3. Results of SM and QPM morphological and physiological trait analyses with Fishers LSD

#	Genotype	RL		RFW		RDW		SL		SFW		SDW		SeedL		SeedDW		VI1		VI2	
		C	T	C	T	C	T	C	T	C	T	C	T	C	T	C	T	C	T	C	T
1	L1	14.83 cdef	3.97 fgh	0.2834 cdefgh	0.0564 ghij	0.0272 abcd	0.0053 fghi	4.25 f	1.02 hi	0.1697 l	0.0401 gh	0.0116 i	0.0042 ghij	19.08 de	4.98 e	0.0388 cd	0.0095 g	1844.4 bc	431.89 g	3.75 ab	0.82 gh
2	L1 QPM 1	16.08 abcde	3.73 h	0.3493 abc	0.0598 fghi	0.0306 abc	0.0054 efghi	4.7 f	1.22 fgh	0.1866 jkl	0.0447 fg	0.0137 hi	0.0045 ghij	20.78 cd	4.95 e	0.0443 abcd	0.0099 fg	2078 abc	462 fg	4.43 ab	0.92 gh
3	L1 QPM 2	16.53 abc	5.32 cde	0.3278 abcd	0.0891 cd	0.0306 abc	0.0074 cd	4.75 f	1.74 bc	0.1701 kl	0.0585 cdef	0.0129 i	0.0053 defgh	21.28 c	7.06 bc	0.0435 abcd	0.0126 def	2057.07 abc	705.83 abcd	4.21 ab	1.26 def
4	L1 QPM 3	14.51 def	5.37 cde	0.2567 defghi	0.0751 def	0.0251 bcd	0.0071 cdef	6.94 cde	1.63 bcde	0.2135 hijkl	0.0440 fg	0.0180 fg	0.0048 efghi	21.45 c	6.99 c	0.0430 abcd	0.0119 ef	2145 abc	652.56 bcde	4.3 ab	1.11 fgh
5	L2	14.32 ef	6.79a	0.3065 abcde	0.1457 a	0.0244 cdef	0.0125 a	7.69 abc	1.18 fghi	0.2184 ghijk	0.0336 gh	0.0171 gh	0.0035 ij	22.00 bc	7.97 ab	0.0414 bcd	0.0159 c	2200.83 abc	743.56 ab	4.14 ab	1.49 cd
6	L2 QPM	14.87 cdef	5.80 bc	0.2962 bcdef	0.1248 b	0.0230 def	0.0103 b	9.08 a	1.32 efgh	0.2587 defg	0.0402 gh	0.0200 cdefg	0.0040 hij	23.94 ab	7.12 bc	0.0429 abcd	0.0144 cde	2154.75 abc	569.33 cdefg	3.86 ab	1.15 efg
7	L3	15.07 bcdef	4.57 efgh	0.3646 ab	0.1395 ab	0.0304 abc	0.0132 a	7.65 bc	1.39 defgh	0.2706 cdef	0.0583 cdef	0.0200 cdefg	0.0064 cde	22.72 abc	5.96 cde	0.0504 ab	0.0196 b	2271.67 ab	556.11 defg	5.04 ab	1.83 b
8	L3 QPM	16.02 abcde	5.34 cde	0.3522 abc	0.1377 ab	0.0315 a	0.0138 a	7.63 bcd	2.18 a	0.3043 abc	0.0902 a	0.0222 cde	0.0092 a	23.64 ab	7.52 ab	0.0537 a	0.0231 a	2048.94 abc	726.61 abc	4.65 ab	2.23 a
9	L4	14.07 f	3.83 gh	0.2142 hij	0.0393 j	0.0184 f	0.0040 i	6.89 cde	1.41 defgh	0.2006 ijkl	0.0420 fg	0.0169 gh	0.0043 fghij	20.96 cd	5.23 de	0.0353 cd	0.0083 g	2025.97 abc	505.89 efg	3.41 b	0.80 h
10	L4 QPM	14.43 def	4.69 defg	0.1924 ij	0.0517 ghij	0.0163 g	0.0048 hi	8.30 ab	1.55 cdef	0.2282 fghij	0.0469 efg	0.0186 defgh	0.0048 fghi	22.73 abc	6.24 cd	0.0349 d	0.0096 g	2273.33 ab	624.17 bcdef	3.49 b	0.96 fgh
11	L5	11.71 g	3.71h	0.1748 j	0.0444 ij	0.0155 g	0.0048 hi	6.59 e	1.26 efgh	0.2385 efghi	0.0499 defg	0.0206 cdefg	0.0057 cdefg	18.30 e	4.97 e	0.0361 cd	0.0105 fg	1708 c	496.83 efg	3.37 b	1.05 fgh
12	L5 QPM	15.21 bcdef	5.53 cd	0.2145 ghij	0.0665 efgh	0.0201 efg	0.0072 cde	9.03 a	1.64 bcde	0.3251 ab	0.0663 bcd	0.0262 ab	0.0072 bc	24.23 a	7.17 bc	0.0464 abc	0.0144 cd	2342.56 abc	717.33 abcd	4.48 ab	1.44 cde
13	L6	15.91 abcde	4.89 cdef	0.2887 cdefgh	0.0685 efg	0.0228 def	0.0069 cdefg	7.75 b,c	1.52 cdefg	0.2340 fghi	0.0484 efg	0.0184 efg	0.0046 fghij	23.65 ab	6.41 cd	0.0412 bcd	0.0115 f	2286.97 ab	576.75 cdefg	3.98 ab	1.03 fgh
14	L6 QPM	16.63 ab	4.71 cdef	0.3788 a	0.0989 c	0.0309 ab	0.0101 b	8.05 b	1.65 bcde	0.2847 bcd	0.0627 cde	0.0225 bcd	0.0059 cdef	24.68 a	6.36 cd	0.0534 a	0.060 c	2468.33 a	614.64 bcdef	5.34 a	1.55 bcd
15	L7	16.14 abcd	6.44 a,b	0.2220 fghij	0.0962 c	0.0195 efg	0.0081 c	6.55 e	2.03 a,b	0.2235 ghij	0.0747 abc	0.0184 efg	0.0068 bcd	22.69 abc	8.47 a	0.0379 cd	0.0149 cd	2193.53 abc	818.44 a	3.67 ab	1.44 cde
16	L7 QPM	17.14 a	6.35 a,b	0.2392 efghij	0.0809 cde	0.0232 def	0.0078 c	7.70 b,c	1.93 a,b,c	0.3304 a	0.0806 ab	0.0292 a	0.0084 ab	24.84 a	8.28 ab	0.0524 ab	0.0162 c	2318.56 ab	827.50 a	4.89 ab	1.62 bc
	Average	15.22	5.06	0.2788	0.0859	0.0243	0.008	7.1	1.54	0.241	0.0551	0.0191	0.0056	22.31	6.6	0.0435	0.0136	2151.12	626.84	4.19	1.29
	LSD$_{0.05}$	1.87	0.89	0.083	0.016	0.00693	0.0016	0.80	0.46	0.04	0.018	0.0037	0.0015	2.16	1.26	0.0114	0.0028	539.6	162.42	1.69	0.33
	Average SM	14.58	4.88	0.2649	0.0843	0.0226	0.0078	6.77	1.4	0.2222	0.0496	0.0176	0.0051	21.34	6.28	0.0402	0.0129	2075.91	589.92	3.91	1.21
	Average QPM	15.71	5.2	0.2897	0.0872	0.0257	0.0082	7.35	1.65	0.2557	0.0593	0.0204	0.006	23.07	6.85	0.0461	0.0142	2238.47	669.48	4.34	1.43

RL-root length, RFW- root fresh weight, RDW-root dry weight, SL-shoot length, SFW-shoot fresh weight, SDW-shoot dry weight, SeedL-seedling length, SeedDW-seedling dry weight, VI1 and VI2-vigor indices, C-control, T-treatment. All different letters in the column designate significant differences at 0.05 probability level.
