# Supplementary material for: Early Sowing Approach for Developing Climate Resilient Maize: Cold Stress Impact on Germination of Adapted Inbred Lines with High Nutritive Value
Source: Plants (Basel). 2025 Aug 15;14(16):2540. doi: 10.3390/plants14162540 (PMC12389089; doi:10.3390/plants14162540)
Supplement: Supplementary file 1 [file plants-14-02540-s001.zip › plants-3789613-supplementary/Table S4 revised.pdf]

Table S4a. Pearsons correlation coefficients between germination parameters and biochemical compounds.

	C					T				
	GP (%)	GI	GE (%)	MGT (days)	GRI (%/day)	GP (%)	GI	GE (%)	MGT (days)	GRI (%/day)
Proteins	0.064	-0.469	-0.364	0.442	-0.397	-0.327	**-0.530**	**-0.599**	**0.501**	-0.228
Tryptophan	0.015	-0.241	-0.111	0.215	-0.182	-0.054	-0.176	-0.302	0.453	-0.097
GA	-0.346	**0.746**	**0.739**	**-0.845**	**0.808**	0.197	**0.699**	**0.695**	**-0.497**	-0.104
PA	-0.010	-0.383	-0.294	0.327	-0.254	**-0.699**	**-0.522**	-0.424	0.215	-0.409
CA	-0.279	0.387	0.428	-0.492	0.442	0.178	0.389	0.377	-0.352	-0.033
p-CoumA	-0.197	**0.672**	**0.704**	**-0.700**	**0.692**	-0.117	**0.535**	**0.676**	**-0.628**	-0.087
FA	-0.272	**0.497**	**0.514**	**-0.583**	**0.603**	0.015	0.427	0.482	-0.476	-0.131
L+Z	0.087	-0.367	-0.338	0.371	-0.327	-0.178	-0.240	-0.152	-0.134	-0.262
β-cryptoxhantin	-0.094	-0.266	-0.240	0.202	-0.229	-0.080	-0.159	-0.064	-0.261	-0.195
β-carotene	0.025	0.216	0.295	-0.170	0.195	0.024	0.246	0.299	-0.342	-0.073
α-T	0.130	-0.136	-0.041	0.199	-0.218	0.045	-0.187	-0.189	0.144	-0.245
β+γ-T	-0.028	0.175	0.124	-0.168	0.135	0.311	0.162	0.155	-0.239	0.147
δ-T	0.273	0.130	0.089	0.038	0.014	0.420	0.088	-0.009	0.073	-0.002

Table of critical values (Degrees of freedom =14):

0.05	0.01
0.497	0.623

C-control, T-treatment, GP-germination percentage, GI-germination index, GE-germination energy, MGT-mean germination time, GRI-germination rate index, GA- gallic acid, PA- protocatechuic acid, CA-caffeic acid, p-CoumA-p-coumaric acid, FA-ferulic acid, L+Z-lutein+zeaxanthin, T-tocopherols.

Table S4b. Pearsons correlation coefficients between morphological and physiological traits and biochemical compounds.

		RL	SL	RFW	SFW	RDW	SDW	SeedL	SeedDW	VI1	VI2
	Proteins	-0.033	-0.453	0.123	-0.418	0.250	-0.411	-0.366	-0.083	-0.331	-0.060
	Tryptophan	**0.622**	-0.145	0.352	0.017	0.366	0.052	0.317	0.353	0.296	0.355
	GA	-0.239	**0.717**	-0.034	**0.758**	-0.137	**0.665**	0.379	0.363	0.212	0.262
	PA	-0.007	-0.391	0.352	**-0.529**	0.381	**-0.601**	-0.302	-0.107	-0.309	-0.107
	CA	-0.002	0.188	0.212	0.368	0.156	0.280	0.141	0.338	0.015	0.257
	p-CoumA	-0.345	**0.526**	0.220	0.365	-0.006	0.253	0.162	0.178	0.072	0.125
C	FA	-0.344	0.314	0.186	0.417	0.093	0.267	0.002	0.273	-0.114	0.203
	L+Z	0.155	-0.394	0.284	-0.226	0.268	-0.301	-0.215	-0.150	-0.153	0.088
	β-cryptoxhantin	-0.379	-0.274	-0.051	-0.042	-0.062	-0.067	-0.471	-0.101	-0.467	-0.114
	β-carotene	0.175	0.059	0.367	0.379	0.285	0.317	0.163	0.469	0.179	0.495
	α-T	-0.147	-0.066	-0.286	-0.059	-0.282	0.026	-0.151	-0.224	-0.078	-0.171
	β+γ-T	0.283	0.211	-0.092	0.159	-0.161	0.183	0.354	-0.007	0.339	-0.025
	δ-T	0.249	0.002	-0.113	0.209	-0.069	0.260	0.172	0.129	0.300	0.223
	Proteins	-0.134	-0.399	-0.222	-0.430	-0.299	-0.415	-0.222	-0.403	-0.297	-0.445
	Tryptophan	0.204	0.140	-0.075	0.053	-0.117	0.000	0.210	-0.091	0.192	-0.087
	GA	0.081	0.223	0.318	0.378	0.461	**0.495**	0.129	**0.561**	0.160	**0.572**
	PA	0.028	-0.491	0.190	**-0.543**	0.048	**-0.569**	-0.111	-0.195	-0.310	-0.318
	CA	-0.198	0.298	0.140	0.416	0.299	0.446	-0.085	0.416	-0.028	0.440
	p-CoumA	0.022	**0.526**	**0.508**	-0.024	**0.588**	0.049	-0.020	0.478	-0.075	0.430
T	FA	-0.312	-0.046	0.265	0.210	0.409	0.323	-0.274	0.451	-0.257	0.434
	L+Z	**-0.502**	-0.310	-0.177	-0.082	-0.128	-0.135	**-0.532**	-0.150	**-0.544**	-0.162
	β-cryptoxhantin	**-0.567**	-0.453	-0.178	-0.057	-0.105	0.026	**-0.631**	-0.074	**-0.613**	-0.078
	β-carotene	-0.335	0.010	0.068	0.335	0.205	0.314	-0.293	0.275	-0.258	0.286
	α-T	-0.063	-0.288	-0.446	-0.243	-0.450	-0.240	-0.132	-0.449	-0.109	-0.422
	β+γ-T	-0.146	0.450	-0.236	0.327	-0.145	0.243	0.000	-0.014	0.096	0.047
	δ-T	-0.198	0.244	-0.459	0.293	-0.348	0.257	-0.099	-0.166	0.043	-0.074

Table S4c. Pearsons correlation coefficients between morphological and physiological traits and germination parameters.

	C					T				
	GP (%)	GI	GE (%)	MGT (days)	GRI (%/day)	GP (%)	GI	GE (%)	MGT (days)	GRI (%/day)
RL	0.064	0.087	0.066	-0.055	0.179	0.020	**0.605**	**0.531**	-0.390	**0.500**
SL	-0.285	**0.822**	**0.805**	**-0.851**	**0.777**	**0.503**	**0.605**	0.443	-0.332	**0.591**
RFW	0.063	0.244	0.298	-0.196	0.388	-0.273	**0.606**	**0.704**	**-0.555**	**0.662**
SFW	-0.471	**0.649**	**0.649**	**-0.800**	**0.741**	**0.504**	**0.685**	**0.507**	-0.398	**0.689**
RDW	0.061	0.067	0.099	-0.046	0.247	-0.193	**0.697**	**0.780**	**-0.623**	**0.757**
SDW	-0.409	**0.571**	**0.563**	**-0.695**	**0.590**	**0.512**	**0.684**	**0.500**	-0.394	**0.697**
SeedL	-0.172	**0.684**	**0.656**	**-0.683**	**0.713**	0.155	**0.576**	**0.567**	-0.418	**0.582**
SeedDW	-0.243	0.471	0.493	**-0.543**	**0.640**	0.059	**0.823**	**0.812**	**-0.647**	**0.875**
VI1	0.326	**0.679**	**0.603**	-0.417	**0.543**	0.443	**0.650**	**0.551**	-0.392	**0.622**
VI2	0.078	0.494	0.490	-0.393	**0.557**	0.244	**0.878**	**0.811**	**-0.637**	**0.910**

RL-root length, SL-shoot length, RFW-root fresh weight, SFW-shoot fresh weight, RDW-root dry weight, SDW-shoot dry weight, SeedL-seedling length, SeedDW-seedling dry weight, VI1 and VI2-vigour indices.
